# Prevalence and factors associated with fertility desires/intentions among individuals in HIV‐serodiscordant relationships: a systematic review of empirical studies

**DOI:** 10.1002/jia2.25241

**Published:** 2019-05-17

**Authors:** Alexandra Martins, Stephanie Alves, Catarina Chaves, Maria C Canavarro, Marco Pereira

**Affiliations:** ^1^ Faculty of Psychology and Education Sciences University of Coimbra Coimbra Portugal

**Keywords:** HIV/AIDS, serodiscordancy, fertility desires/intentions, prevalence, factors, systematic review

## Abstract

**Introduction:**

Better knowledge about fertility desires/intentions among HIV‐serodiscordant partners who face unique challenges when considering childbearing may be helpful in the development of targeted reproductive interventions. The aim of this systematic review was to synthesize the published literature regarding the prevalence of fertility desires/intentions and its associated factors among individuals in HIV‐serodiscordant relationships while distinguishing low‐ and middle‐income countries (LMIC) from high‐income countries (HIC).

**Methods:**

A systematic search of all papers published prior to February 2017 was conducted in four electronic databases (PubMed/MEDLINE, PsycINFO, Web of Science and Cochrane Library). Empirical studies published in peer‐reviewed journals with individuals in HIV‐serodiscordant relationships assessing the prevalence of fertility desires/intentions and/or the associated factors were included in this systematic review. This review adhered to Preferred Reporting Items for Systematic Reviews and Meta‐Analyses (PRISMA) guidelines.

**Results and discussion:**

After screening 1852 references, 29 studies were included, of which 21 were conducted in LMIC and eight in HIC. A great variability in the prevalence of fertility desires/intentions was observed in LMIC (8% to 84% (one member of the dyad included)). In HIC, the results showed a smaller discrepancy between in the prevalence (32% to 58% (one member of the dyad included)); the prevalence was higher when the couple was the unit of analysis (64% to 73%), which may be related to the fact that all these studies were conducted in the context of assisted reproduction. Few studies examined the factors associated with fertility desires/intentions, and all except one were conducted in LMIC. Individuals (e.g. number of children), couple‐level (e.g. belief that the partner wanted children) and structural factors (e.g. discussions with health workers) were found to be associated.

**Conclusions:**

The results of this systematic review suggest that many individuals in HIV‐serodiscordant relationships have fertility desires/intentions, although the prevalence is particularly heterogeneous in LMIC in comparison to HIC. Well‐known factors such as younger age and a fewer number of living children were consistently associated with increased fertility desires/intentions. Different couple‐level factors emerged, reflecting the importance of considering both the individual and the couple. However, further studies that specifically focus on the dyad as the unit of analysis are warranted.

## 1 Introduction

The improved life expectancy and stabilized HIV infection prevalence in many countries suggest that the number of HIV‐serodiscordant couples (i.e. one member of the couple is living with HIV and the other is not) is likely to continue to increase 1. Although data about the prevalence of serodiscordancy in high‐income countries (HIC) have been scarcely reported 2, data from African countries suggest high rates of serodiscordant relationships (e.g. at least two‐thirds of couples living with HIV are in five sub‐Saharan African countries) 3, 4. At the beginning of the HIV epidemic, as indicated by the Centers for Disease Control and Prevention, couples with an partner living with HIV were discouraged from considering childbearing because of the poor prognosis of those infected and the few options to reduce the risk of HIV transmission 5. Currently, these couples are planning their futures together, which may include the desire and intention to have biological children 6.

Regarding reproductive issues, individuals in serodiscordant relationships may be an important population 7, 8, 9. Serodiscordant couples face the unique challenge of minimizing the risk of HIV transmission to both the uninfected partner and any offspring 10. Nevertheless, many safer conception strategies currently exist that may be compatible with their fertility desires/intentions 11. One important strategy has been the uptake of antiretroviral therapy (ART) to suppress HIV viraemia. The UNAIDS have recently endorsed the concept of *Undetectable = Untransmittable*, given the strong scientific consensus that people living with HIV (PLWH) who are taking effective ART and whose level of HIV is suppressed to undetectable levels cannot transmit HIV sexually to their partners 12, 13, 14, 15. Many other strategies exist, which include reserving condomless sex for days with peak fertility, home manual insemination, medical male circumcision and pre‐exposure prophylaxis (PrEP) to protect the partner living without HIV 11, 16, 17. Medically assisted reproduction is also available in many developed countries, although the costs and limited accessibility, particularly in resource‐limited settings, make this unreachable for most serodiscordant couples 17, 18.

Both fertility decision‐making and safer conception interventions should ideally involve both partners of the serodiscordant relationship 19, 20. However, some challenges cannot be overlooked, such as gender power dynamics and communication between partners, including (non‐)disclosure of HIV status. Unequal gender power dynamics within sex‐opposite couples have led men, regardless of who is living with HIV, to play a dominant role in decisions about fertility, determining if, how and when to conceive 9, 20. For example, Matthews *et al*. 19, in their study with PLWH on ART who reported a partner living without HIV or a partner with unknown serostatus, suggested that many couples made incorrect assumptions about their partner's desires, had disparate understandings about HIV transmission and disagreed on the acceptable level of HIV risk to meet reproductive goals. This study also reinforced the importance of assessing and supporting disclosure of HIV status between partners, which is required for effective use of some safer conception options, as timed intercourse 9.

It is critical to understand fertility desires/intentions in the continuum of care supporting reproductive health 21, so that individuals in serodiscordant relationships can be assisted in conceiving safely in the future, delaying or limiting unwanted pregnancies using effective contraception options (including for those who do not consider having children) 22. However, much of the research on fertility desires/intentions has focused on PLWH as a whole (or, more specifically, women living with HIV (WLWH)), with particular attention to sub‐Saharan Africa, where the HIV prevalence is high and modern contraceptive access and use are low 22, 23. Concerning PLWH, studies conducted after the introduction of combination therapies in 1996 have suggested that a substantial proportion would like/expect to have children. However, this prevalence varied greatly by country and by study 24, 25, 26.

Among PLWH/WLWH, but not specifically in serodiscordant relationships, abundant research has been interested in identifying the factors associated with fertility desires/intentions. One systematic review 27, and despite some divergent results in the individual studies, indicated that younger PLWH and those under family and sociocultural pressure, from a particular cultural/ethnic background, with fewer/no children, on ART, who felt healthier and who have lost children to HIV/AIDS may be more likely to consider having children. A meta‐analytic review conducted by Berhan and Berhan 28 demonstrated that the fertility desire of PLWH was highest among young and childless individuals. A recent meta‐analysis 29 concluded that none of the factors examined (availability of highly active ART; time since ART became widely available; cohabiting status) had influence on the fertility desire of WLWH. These reviews did not analyse the prevalence of fertility desires/intentions, although they showed a great diversity of associated factors, suggesting the complexity of this issue. Also, these studies did not consider only those in an intimate relationship; however, it may be important to analyse this association in more specific sub‐populations, such as couples with a partner living with HIV. Moreover, the aggregation of outcomes from studies with different economies and samples in the first two reviews may complicate the comparability and synthesis of the findings 2.

In this review, we adopted the definitions of fertility desires and intentions proposed by the traits‐desires‐intentions‐behaviour (T‐D‐I‐B) theoretical framework 30, 31. Fertility desires reflect a wish to achieve a goal through some sort of action (i.e. they represent what the individual would like/want to do about having/not having a child based on his/her feelings given no situational constraints), whereas fertility intentions involve a specific decision to pursue an actionable goal with an associated commitment and a plan for implementing the decision 32. However, these terms are often used interchangeably, due to inadequate or poor construct definition/operationalization, and are rarely measured separately. Because it is not always possible to capture these variations when interpreting the studies, we used the general term fertility desires/intentions to refer to any of the constructs. Regarding the associated factors, we used a categorization based on the social ecological framework developed by Crankshaw *et al*. 33 for understanding HIV risk behaviour in the context of supporting serodiscordant couples’ fertility goals. This categorization includes: individual factors (e.g. ART adherence), couple‐level factors (e.g. couple's communication, gender power) and the structural domain (e.g. cultural context, health system). This framework is particularly useful to identify which factors are most likely to influence the fertility desires/intentions at each level of the social ecological approach as well as to develop potential interventions across multiple‐levels to address the different challenges faced by couples 33, 34.

This systematic review aimed to comprehensively review and synthesize the literature regarding the prevalence of the desire/intention to have children and the factors associated with fertility desires/intentions among individuals in serodiscordant relationships, distinguishing low‐ and middle‐income countries (LMIC) from HIC. This focus on individuals in serodiscordant relationships is important because within any couple's relationship, there is almost inevitably a strong reciprocal influence between fertility desires/intentions as well as a combined effect on their conjoint instrumental behaviours 35. Because different resource levels contribute to distinct socio‐structural environments requiring separate consideration 2, this review differentiates LMIC from HIC, being the first to do so.

## 2 Methods

We performed a systematic literature search according to the guidelines of the Preferred Reporting Items for Systematic Reviews and Meta‐Analyses (PRISMA) statement 36.

### 2.1 Data sources and search strategy

The first author conducted a systematic search of all papers published prior to 21 February 2017, in four electronic databases: PubMed/MEDLINE, PsycINFO, Web of Science™ Core Collection and Cochrane Library – Cochrane Central Register of Controlled Trials (CENTRAL). The Cochrane Database of Systematic Reviews was also searched for existing reviews on the topic. Three basic sets of search terms were used to identify records related to the condition of interest (HIV/AIDS), the outcome of interest (fertility desires/intentions) and the participants to be included (individuals in serodiscordant relationships). The detailed search strategy used for searching the PsycINFO database is presented (see Additional File). This search strategy was used for all databases, with slight adaptations to fit different web interfaces. The Medical Subject Headings terms were used in PubMed/MEDLINE and Cochrane Library, and the Subject Heading in PsycINFO. Secondary reference searching was also conducted on the reference lists of the articles included in this review and in any systematic reviews/meta‐analyses relevant to the research question.

### 2.2 Eligibility criteria and study selection

This systematic review involved studies with the following inclusion criteria: (1) studies with individuals in serodiscordant relationships, including one or both members of the couple, in which the frequency of individuals in these relationships must be reported. Serodiscordant couples/partners were considered sexual partnerships in which one member is living with HIV (index partner) and the other is living without HIV or his/her HIV status is unknown. Partners of any sexual orientation were eligible; (2) studies assessing the prevalence of fertility desires/intentions and/or the associated factors, reporting at least one finding of interest. The eligibility criteria required that data on fertility desires/intentions were provided by the individuals in serodiscordant relationships, assessed before the time of conception and were an outcome of a study when assessing the associated factors; (3) empirical studies (quantitative, mixed methods or qualitative); and (4) studies published in peer‐reviewed journals. The exclusion criteria are detailed at Additional File.

After removing duplicates, the first author screened the titles and abstracts of all retrieved records and applied the eligibility criteria. Irrelevant records were discarded, and the full‐text was retrieved for all potentially relevant or unclear articles. The full‐texts were assessed for inclusion by the first author. Any uncertainty related to the inclusion of a study was resolved by discussion with the last author. If any clarification or further information was required, the corresponding authors of the original studies were contacted. When those articles remained unclarified, we conducted the systematic review without analysing these studies.

### 2.3 Data collection and data items

A data extraction form was developed using the Data Extraction Template for Included Studies 37 as a guide. The data extraction form was pilot‐tested for feasibility and comprehensiveness with five studies and refined accordingly. The first author assessed each full‐text article and extracted the required data, and the second author checked the extracted data. Disagreements were resolved by discussion between these authors. Any disagreement was resolved by discussion with the last author.

Extracted information included: (1) authors and year of publication; (2) country(ies) where the research was conducted and year(s) of data collection; (3) study design; (4) sample/subsample size; (5) members of the dyad; (6) sex of the index partner; (7) method of assessment of fertility desires/intentions; (8) relevant findings: prevalence of fertility desires/intentions and/or associated factors among individuals in serodiscordant relationships. The studies were grouped according to the World Bank country classification scheme, distinguishing LMIC (Table 1) from HIC (Table 2). When data from the same study were reported in different journal articles, priority was given to the article that best answered our research question.

**Table 1:** Summary of included studies conducted in LMIC

Study (year) [Reference]	Country and year(s) of data collection	Study design	Sample/subsample size	Members of the dyad	Sex of the index partner	Method of assessment of fertility desires/intentions	Relevant findings: Prevalence	Relevant findings: Factors
Antelman *et al*. (2015) 24	Kenya Namibia Tanzania 2009 to 2010	QT, CS	629 of the PLWH reported an HIV–partner (351 HIV+ women; 278 HIV+ men) 1053 of the PLWH reported an HIV? partner (779 HIV+ women; 274 HIV+ men)	One	Among PLWH with an HIV– partner, 56% (351/629) were HIV+ women Among PLWH with an HIV? partner, 74% (779/1053) were HIV+ women	Women were asked if they desired a pregnancy in the next six months and men if they desired their partner to become pregnant in the same period	19% (120/629) of PLWH with HIV– partner reported to desire pregnancy (self or partner) in the next six months 15% (155/1053) of PLWH with HIV? partner reported to desire pregnancy (self or partner) in the next six months	NA
Beyeza‐Kashesya *et al*. (2010) 47	Uganda 2007	QT, CS	114 serodiscordant couples (228 individuals)	Both	In 52% (59/114) of the couples, the HIV+ partner was the man	Personal (or partner's believed) desire to have children was assessed and recorded as: *Not at all*;* I don't know*;* Maybe I/he/she want(s)*;* Definitely I/he/she want(s)*. The responses *Maybe I/he/she want(s)* and *Definitely I/he/she want(s)* expressed the desire to have children. A follow‐on question asked about the number of children the participant planned to have (Individual/Separate assessment)	Desire of individuals: 59% (135/228) of participants reported to desire to have children sometime in the future Considering the sex of the index partner: 64% (70/110) participants in +woman couples 55% (65/118) participants in +man couples Plans of individuals: Of the participants who reported to desire to have children, 97% reported that they planned to have a definite number of children	Factors associated with increased desire considering the sex of the index partner: +woman couples: ‐ Younger age (30 years or less) (AOR 3.33 (95% CI 1.03, 10.8), *p *=* *0.045) ‐ Having three or fewer living children (OR 4.89 (95% CI 1.95, 12.3), *p *<* *0.001) ‐ The belief that their partner wants children (AOR 26.3 (95% CI 7.89, 87.6), *p *<* *0.001) ‐ Pressure from relatives for the couple to have a baby (AOR 6.77 (95% CI 1.12, 21.7), *p *<* *0.01) ‐ Wanting serostatus to remain a secret (OR 2.36 (95% CI 1.02, 5.49), *p *=* *0.022) ‐ Not having disclosed HIV status to relatives (OR 0.38 (95% CI 0.16, 0.90), *p *=* *0.039) ‐ Having held discussions with the partner on when to get pregnant (OR 3.78 (95% CI 1.46, 9.75), *p *=* *0.003) +man couples: ‐ Younger age (30 years or less) (OR 2.59 (95% CI 1.13, 5.94), *p *=* *0.020) ‐ Having three or fewer living children (OR 5.60 (95% CI 2.34, 13.4), *p *<* *0.001) ‐ The belief that their partner wants children (AOR 24.0 (95% CI 9.15, 105.4), *p *<* *0.001) ‐ Possessing the knowledge that ART is more than 70% effective (AOR 3.66 (95% CI 1.15, 11.7); *kp *=* *0.029) ‐ Pressure from relatives for the couple to have a baby (OR 3.45 (95% CI 1.55, 7.70), *p *<* *0.01) ‐ Not having had discussions with health workers about contraception and HIV (AOR 0.29 (95% CI 0.08, 0.96), *p *=* *0.042) Factors not associated with desire considering the sex of the index partner: +woman couples: ‐ Being on ART (self or partner) ‐ Possessing the knowledge that ART is more than 70% effective ‐ Discussing with health workers about contraception and HIV ‐ Discussing with health workers about pregnancy and HIV +men couples: ‐ Being on ART (self or partner) ‐ Wanting serostatus to remain a secret ‐ Disclosing HIV status to relatives ‐ Discussing with the partner on when to get pregnant ‐ Discussing with health workers about pregnancy and HIV
Demissie *et al*. (2014) 56	Ethiopia 2013	MX, CS	60 of PLWH reported to have an HIV– partner	One	NR	“Did you have fertility desire?” (binary response choices: *Yes*/*No*)	33% (20/60) of PLWH with HIV– partner reported fertility desire	NA
Guthrie *et al*. (2010) 1	Kenya 2007 to 2009	QT, CO	454 women in serodiscordant couples (293 HIV+ and 161 HIV–)	One	Among women in serodiscordant couples, 65% (293/454) were HIV+ women	NR	46% (n = 204) women in serodiscordant couples reported to desire additional children Desire of HIV+ and HIV– women: 48% (n = 137) HIV+ 42% (n = 66) HIV–	NA
Gutin *et al*. (2014) 24	Uganda 2007	QT, CS	47 postnatal WLWH reported an HIV– partner	One	Women (n = 47)	Asked if they planned to have more children in the future and whether they were currently in a sexual relationship	43% (20/47) of WLWH with HIV– partner reported to desire more children in the future	NA
Gyimah *et al*. (2015) 70	Ghana 2012	QT, CS	75 WLWH reported an HIV– partner 61 WLWH reported an HIV? partner	One	Women (n = 136)	NR	61% (46/75) of the WLWH with HIV– partner reported to desire to have a child 64% (39/61) of the WLWH with HIV? partner reported to desire to have a child	NA
Iliyasu *et al*. (2009) 61	Nigeria 2007	QT, CS	21 PLWH reported to have an HIV– partner 49 PLWH reported to have an HIV? partner	One	NR	NR	67% (14/21) of PLWH with HIV– partner reported desire more children 43% (21/49) of PLWH with HIV? partner reported desire more children	NA
Jose *et al*. (2016) 52	India 2012 to 2014	QT, CS	77 PLWH reported to have an HIV– partner 12 PLWH reported to have an HIV? partner	One	NR	Asked if they would like to have children in the future (binary response choices: *Yes*/*No*)	29% (22/77) of PLWH with HIV– partner reported fertility desire 8% (1/12) of PLWH with HIV? partner reported fertility desire	NA
Kuete *et al*. (2016) 49	Cameroon 2014	QT, CS	94 pregnant WLWH living with HIV– partners	One	Women (n = 94)	Although a questions(s) to specifically address the ideal number of children and future fertility was(were) not described, an operational definition for future fertility was presented: number of future pregnancies/couple's plans regarding future pregnancies	84% (79/94) of the WLWH living with HIV– partners reported an ideal number of children (one or more) 81% (76/94) of the WLWH living with HIV– partners reported future fertility	Factor associated with increased future fertility: ‐ Fewer number of living children (r* *=* *−0.22, *p *=* *0.036)
Matthews *et al*. (2013) 58	South Africa 2010	QL, CS	50 PLWH with an HIV– or HIV? partner (30 HIV+ women with recent pregnancy and 20 HIV+ men)	One	Among PLWH with an HIV– or HIV? partner, 60% (30/50) were HIV+ women	NR	44% (22/50) of PLWH with an HIV– or HIV? partner reported desire for child in future Desire of HIV+ women and men: 27% (8/30) HIV+ women 70% (14/20) HIV+ men	Category: “I. Reproductive decision‐making” Factor associated with decreased desire: Particularly for men: ‐ Higher number of living children (Illustrative quote: “Most male participants expressed a desire for children in the future; those who did not desire children in the future reported at least one living child”)
Melaku *et al*. (2014) 63	Ethiopia 2013	QT, CS	85 WLWH reported an HIV– partner	One	Women (n = 85)	“Would you like to have children in the future?” (dichotomized into “Had no desire” if a woman answered *No*, and “Had fertility desire” if she answered *Yes*)	59% (50/85) of WLWH with HIV– partner reported fertility desire	NA
Melka *et al*. (2014) 71	Ethiopia 2012	QT, CS	57 WLWH reported an HIV– partner	One	Women (n = 57)	NR	63% (36/57) of WLWH with HIV– partner reported fertility desire	NA
Mujugira *et al*. (2013) 50	Kenya Uganda 2011	QT, CS	571 serodiscordant couples (1142 individuals)	Both	In 64% (368/571) of the couples, the HIV+ partner was the woman	Questions about the number and timing of additional children were included in the questionnaire, although it is not clear if these items measured desired additional children/fertility intentions (Individual/Separate assessment)	Intentions of couples (only one member or both members): For 45% (257/571) of the couples one or both members reported fertility intentions Intentions of both members of the couples: For 21% (121/571) of the couples both members reported fertility intentions Intentions of HIV+ partners: 33% (190/571) HIV+ Intentions of HIV+ women and men: 36% (134/368) HIV+ women 28% (56/203) HIV+ men	Factors associated with increased intentions among HIV+ partners: ‐ Expressing interest in early ART (i.e. at CD4 counts >350 cells/μL) for HIV‐1 prevention (AOR 1.83 (95% CI 1.12, 299), *p *=* *0.02) ‐ Younger age (<25 years) (25 to 34 years old: AOR 4.97, (95% CI 1.96, 12.63), *p *<* *0.01; 18 to 24 years old: AOR 10 to 63 (95% CI 3.68, 30.70), *p *<* *0.001) ‐ Being male (AOR 1.65 (95% CI 1.00, 2.73), *p *=* *0.05) ‐ Lack of children with their partner (AOR 2.54 (95% CI 1.42, 4.53) *p *=* *0.002) ‐ Having unprotected sex in the prior month (AOR 1.67 (95% CI 1.00, 2.77), *p *=* *0.05) Factors not associated with intentions among HIV+ partners: ‐ Education ‐ Partnership duration
Muldoon *et al*. (2017) 60	Uganda 2009 to 2011	QT, CS	409 serodiscordant couples (818 individuals)	Both	In 58% of the couples, the HIV+ partner was the man	“Do you want to have more biological children?” (response choices: *Yes*/*No*/*Don't know*/*Not applicable*) (Individual/Separate assessment)	Desire of individuals: 28% (225/818) of the participants reported to want more biological children Desire of couples (only one member or both members): For 39% (158/409) of the couples one or both members reported to want more biological children Desire of both members of the couples: For 16% (67/409) of the couples both members reported to want more biological children Desire of women and men: 23% (93/409) women 32% (132/409) men	NA
Myer *et al*. (2007) 57	South Africa 2005	MX, CS	39 PLWH reported to have an HIV– partner (27 HIV+ women; 12 HIV+ men)	One	Among PLWH with an HIV– partner, 69% (27/39) were HIV+ women	“Do you want to have children, or more children, in the future?”	39% (15/39) of PLWH with HIV– partner reported pregnancy desire Desire of HIV+ women and men: 44% (12/27) HIV+ women 25% (3/12) HIV+ men	NA
Ndlovu (2009) 59	Zimbabwe 2005	QL, CS	2 serodiscordant couples (four individuals)	Both	In both couples, the HIV+ partner was the woman (n = 2)	NR (Individual/Separate assessment)	Desire of individuals: 50% (2/4) of the participants reported to desire to have children Desire of both members of the couples: For 50% (1/2) of the couples both members reported desires to have children Desire of women (the HIV+ partners) and men: 50% (1/2) HIV+ women 50% (1/2) men Intention of individuals: 25% (1/4) of the participants reported to intent to have children Intention of both members of the couples: For neither of the couples both members reported intentions to have children Intention of women (the HIV+ partners) and men: None of the HIV+ women reported intention 50% (1/2) men	NA
Nóbrega *et al*. (2007) 54	Brazil 2004	QT, CS	34 WLWH reported to have an HIV– partner	One	Women (n = 34)	NR	65% (22/34) of the WLWH with a HIV– partner reported to have the desire to have a child	NA
Okome‐Nkoumou *et al*. (2015) 62	Gabon 2010 to 2011	QT, CS	136 PLWH reported to have an HIV– partner	One	NR	NR	81% (110/136) of the PLWH with HIV– partner reported to desire to have children	NA
Paiva *et al*. (2007) 55	Brazil 1999 to 2000 (women) 2001 to 2002 (men)	QT, CS	284 PLWH reported to have an HIV– partner (177 HIV+ women; 107 HIV+ men) Eight HIV+ men reported to have an HIV? partner	One	Among PLWH with an HIV– partner, 62% (177/284) were HIV+ women All participants with an HIV? partner were HIV+ men	NR	36% (103/284) of the PLWH with HIV– partner reported to desire to have children 63% (5/8) of HIV+ men with HIV? partner reported to desire to have children	NA
Rispel *et al*. (2011) 51	South Africa Tanzania 2008	MX, CS	36 serodiscordant couples (72 individuals)	Both	In 64% (23/36) of the couples, the HIV+ partner was the woman	NR (Individual/Separate and couples’ assessment)	49% (33/67) of the participants reported to want (additional) child/children	Category: “Desire for children and reproductive decisions” Couples’ intentions were influenced by: ‐ Fear of infecting the HIV– partner ‐ Conflicting desires of the two partners ‐ Medical professional advice ‐ The lack of availability and affordability of alternatives to condomless heterosexual vaginal intercourse ‐ Not having any children Couples’ intentions were not influenced by: ‐ Being on ART
Venkatesh *et al*. (2011) 53	India 2008	QT, CS	103 PLWH who reported to be in a serodiscordant relationship (HIV– partner) (20% HIV+ women; 80% HIV+ men)	One	Among PLWH with a serodiscordant partner, 80% were HIV+ men	Although a question(s) to address fertility intent was(were) not described, fertility intent was defined as whether the participant was interested in having a child	62% of PLWH in a serodiscordant relationship reported to want to have a child	NA

All values presented as a percentage were rounded to units. Antiretroviral treatment (ART). Data not reported (NR). Partner living without HIV (HIV−). Partner living with HIV (HIV+). HIV unknown status (HIV?). Members of the dyad: The study included only one member of the dyad (One); and the study included both members (Both). Method of assessment of fertility desires/intentions: The research question that specifically addresses fertility desires/intentions for our sample/subsample of individuals in serodiscordant relationships. For studies that included both members of the dyad, a note about whether the assessment was individual/separate or of the couple was also reported. Not applicable (NA). For the item Relevant findings: Factors, “Not applicable (NA)” was used when the study did not aim to assess the factors associated with fertility desires/intentions or the design of the study (e.g. the factors were analysed for the total sample of PLWH) did not allow to answer this question. People living with HIV (PLWH): Men and women living with HIV or AIDS. Positive‐man couples (+man couples): Couples in which the man is the index partner. Positive‐woman couples (+woman couples): Couples in which the woman is the index partner. Sample/subsample size: Number of serodiscordant couples or participants in serodiscordant relationships. Sex of the index partner: Frequency and/or rate of the sex of the partner living with HIV. In the subsample of PLWH in a serodiscordant relationship, the number of women or men living with HIV was divided by the total number of participants in the subsample. Study design: Quantitative data (QT); qualitative study (QL); mixed methods study (MX); cross‐sectional study (CS); cohort study (CO). Women living with HIV (WLWH): Only women living with HIV or AIDS.

**Table 2:** Summary of included studies conducted in HIC

Study (year) [Reference]	Country and year(s) of data collection	Study design	Sample/subsample size	Members of the dyad	Sex of the index partner	Method of assessment of fertility desires/intentions	Relevant findings: Prevalence	Relevant findings: Factors
Finocchario ‐Kessler *et al*. 25	US Year(s) of data collection NR	QT, CS	121 WLWH reported to have an HIV– partner (n = 103) or HIV? partner (n = 18)	One	Women (n = 121)	“How many children do you want to have in the future?” Responses greater than zero denote desires to have a future child	55% (67/121) of WLWH with HIV– partner or HIV? partner reported to desire child in the future	NA
Gosselin & Sauer (2011) 6	US 2002 to 2009	QT, CS ‐ RCR	143 serodiscordant couples (286 individuals)	Both	Men (n = 143)	“Would you like to have more children in the future if attempts to conceive are successful?” (binary response choices: *Yes*/*No*) (Individual/Separate assessment)	Desire of both members of the couples (+man couples): For 70% (n = 94) of the couples both members reported to want to have children in the future, even after one successful cycle of fertility treatment Desire of women and men (the HIV+ partners): 73% (n = 99) women 72% (n = 98) HIV+ men	Factors associated with increased couple's desire: ‐ Younger age (OR 0.86 (95% CI 0.75 to 0.99), *p *=* *0.04) ‐ Not having children together (OR 10.03 (95% CI 2.27 to 44.26), *p *<* *0.01) ‐ Beginning the relationship after the male partner had already been diagnosed (OR 6.19 (95% CI 1.27 to 30.25), *p *=* *0.02) ‐ Shorter relationship length (OR 0.988; (95% CI 0.978 to 0.998), *p *=* *0.02) Factors not associated with couple's desire: ‐ Total discussion score (the total score of these items: discussion of the risk of horizontal and vertical transmission; discussion about the partner's potential premature death; discussion of third‐party parenting in the event of his death; discussion about using partner's banked sperm in event of his death)
Haddad *et al*. 68	US 2013 to 2014	QT, CS	102 WLWH reported an HIV– partner 38 WLWH reported an HIV? partner	One	Women (n = 140)	“Do you want or plan to have more children (at any time in the future)?” Desire for future children was defined as reporting *Yes* to the question	32% (33/102) of WLWH with HIV– partner reported to desire for future children 50% (19/38) of WLWH with HIV? partner reported to desire for future children	NA
Klein *et al*. 65	US 1999 to 2001	MX, CS	50 serodiscordant couples (100 individuals)	Both	Men (n = 50)	“If IVF‐ICSI is successful (healthy child and no viral transmission), would you undergo another cycle to have more children?” (Individual/Separate assessment)	Desire of individuals (in +man couples): 66% (66/100) of participants reported that they would like to pursue a second child through IVF‐ICSI if the method resulted in a healthy first child Desire of women and men (the HIV+ partners): 66% (33/50) women 66% (33/50) HIV+ men	NA
Mindry *et al*. 66	US year(s) of data collection NR	MX, CS	26 PLWH reported to have an HIV– partner (n = 20) or HIV? partner (n = 6)	One	NR	Fertility desires were measured in response to a two‐part question: “Do you wish to have a/another child, either now or in the future?” (binary response choices: *Yes*/*No*), with a follow‐up question to those who responded *No*: “Would your desire to have a/another child change if you knew you could have a child with limited risk of transmitting HIV to your partner and the child?” (binary response choices: *Yes*/*No*). Respondents who answered *Yes* to one of the questions above were categorized as having fertility desires	58% (15/26) of PLWH with HIV– partner or HIV? partner reported fertility desires	NA
Panozzo *et al*. 64	Switzerland 2000 to 2001	QT, CS	43 PLWH reported to be in a serodiscordant relationship (HIV– partner)	One	NR	NR	42% (18/43) PLWH in a serodiscordant relationship reported a current strong desire for children Number of participants with a deferred desire: NR	NA
Peña *et al*. 67	US 1997 to 2002	QT, CO	11 serodiscordant couples (22 individuals)	Both	Men (n = 11)	“If successful pregnancy, would consider more children?” (Individual/Separate assessment)	64% of women and 73% of men (the HIV+ partners) reported they would consider more children, if successful pregnancy	NA
Rhodes *et al*. 69	US 2012 to 2014	QT, CS	61 WLWH reported to have HIV– partner Six WLWH reported to have HIV? partner	One	Women (n = 67)	“Do you desire to have children in the future?” determined future reproductive desire (binary response choices: *Y*es/*N*o)	44% (27/61) of the WLWH with HIV– partner reported desire for future children 33% (2/6) of the WLWH with HIV? partner reported desire for future children	NA

All values presented as a percentage were rounded to units. Antiretroviral treatment (ART). Data not reported (NR). Partner living without HIV (HIV–). Partner living with HIV (HIV+). HIV unknown status (HIV?). *In vitro* fertilization‐intracytoplasmic sperm injection (IVF‐ICSI). Members of the dyad: The study included only one member of the dyad (One); and the study included both members (Both). Method of assessment of fertility desires/intentions: The research question that specifically addresses fertility desires/intentions for our sample/subsample of individuals in serodiscordant relationships. For studies that included both members of the dyad, a note about whether the assessment was individual/separate or of the couple was also reported. Not applicable (NA). For the item Relevant findings: Factors, “Not applicable (NA)” was used when the study did not aim to assess the factors associated with fertility desires/intentions or the design of the study (e.g. the factors were analysed for the total sample of PLWH) did not allow to answer this question. People living with HIV (PLWH): Men and women living with HIV or AIDS. Positive‐man couples (+man couples): Couples in which the man is the index partner. Sample/subsample size: Number of serodiscordant couples or participants in serodiscordant relationships. Sex of the index partner: Frequency and/or rate of the sex of the partner living with HIV. In the subsample of PLWH in a serodiscordant relationship, the number of HIV‐infected women or men was divided by the total number of participants in the subsample. Study design: Quantitative data (QT); mixed methods study (MX); cross‐sectional study (CS); cohort study (CO); retrospective chart review (RCR). United States of America (US). Women living with HIV (WLWH): Only women living with HIV or AIDS.

### 2.4 Assessment of risk of bias

For quantitative studies, the risk of bias was assessed using criteria developed from Sanderson *et al*.'s 38 systematic review and the US National Heart, Lung, and Blood Institute Quality Assessment Tool for Observational Cohort and Cross‐Sectional Studies (individual criteria presented in Table 3) 39. For mixed methods studies, we used the criteria developed from the Mixed Methods Appraisal Tool (individual criteria in Table 4) 40. Regarding qualitative studies, the risk of bias was assessed using the criteria developed from the Critical Appraisal Skills Program checklist (Table 5) 41. For all study types, the rating system was based on a system previously used 42: if >60% of the criteria on the checklist were met (strong quality); 40% to 60% (moderate quality); and <40% (poor quality). Risk of bias was appraised independently by the first and second authors. Discrepancies were resolved by discussion to reach consensus. Inter‐rater agreement was calculated with Cohen's Kappa coefficient, considering *k *< 0.00 as poor, *k *≤ 0.20 as slight, *k *≤ 0.40 as fair, *k *≤ 0.60 as moderate, *k *≤ 0.80 as substantial and *k *> 0.81 as almost perfect agreement 43. The percentage of agreement was calculated to triangulate the *k* statistic, which has the limitation of being sensitive to cell size. No study was excluded on the basis of the assessment of risk of bias, which was used to improve our understanding of the relative strengths and weaknesses of the evidence.

**Table 3:** Risk of bias assessment of included quantitative studies

Study (year) [Reference]	Objective clearly stated^a^	Study population clearly defined and eligibility criteria described^b^	Representative sample^c^	Participation rate of eligible individuals ≥50%^d^	Exposure assessed prior to outcome measurement^e^	Appropriate outcome measures for the outcome of interest^f^	Loss to follow‐up after baseline ≤20%^g^	Methods to control confounding^h^	Rating
Antelman *et al*. (2015) 24	Y	Y	Y^i^	Y^i^	N	Y	NA	Y	Strong (85.7%)
Beyeza‐Kashesya *et al*. (2010) 47	Y	Y	N	Y	N	Y	NA	Y	Strong (71.4%)
Finocchario‐Kessler *et al*. (2010) 25	Y	Y	N	NR	N	Y	NA	Y	Moderate (57.1%)
Gosselin & Sauer (2011) 6	Y	N^j^	N	NA	N	Y	NA	NR	Poor (33.3%)
Guthrie *et al*. (2010) 1	Y	Y	NR	NR	Y	NR	NR	Y	Moderate (50%)
Gutin *et al*. (2014) 22	Y	Y	N	Y	N	Y	NA	Y	Strong (71.4%)
Gyimah *et al*. (2015) 70	Y	Y	N	Y	N	NR	NA	Y	Moderate (57.1%)
Haddad *et al*. (2016) 68	Y	Y	NR	NR	N	Y	NA	Y	Moderate (57.1%)
Iliyasu *et al*. (2009) 61	Y	Y	Y	Y	N	NR	NA	Y	Strong (71.4%)
Jose *et al*. (2016) 52	Y	Y	N	NR	N	Y	NA	NR	Moderate (42.9%)
Kuete *et al*. (2016) 49	Y	Y	N	Y^k^	N	NR	NA	NR	Moderate (42.9%)
Melaku *et al*. (2014) 63	Y	Y	Y	Y	N	Y	NA	Y	Strong (85.7%)
Melka *et al*. (2014) 71	Y	N^j^	Y	Y	N	NR	NA	Y	Moderate (57.1%)
Mujugira *et al*. (2013) 50	Y	Y	Y^l^	Y^l^	N	NR	NA	Y	Strong (71.4%)
Muldoon *et al*. (2017) 60	Y	Y^m^	N^m^	Y	N	Y	NA	NR	Moderate (57.1%)
Nóbrega *et al*. (2007) 54	Y	Y	NR	Y	N	NR	NA	Y	Moderate (57.1%)
Okome‐Nkoumou *et al*. (2015) 62	Y	Y	N	Y	N	NR	NA	NR	Moderate (42.9%)
Paiva *et al*. (2007) 55	Y	N	N	NR	N	NR	NA	Y	Poor (28.6%)
Panozzo *et al*. (2003) 64	Y	N	N	N	N	NR	NA	NR	Poor (14.3%)
Peña *et al*. (2003) 67	Y	Y	N	NR	Y	Y	NR	NR	Moderate (50%)
Rhodes *et al*. (2016) 69	Y	Y	N	Y	N	Y	NA	Y	Strong (71.4%)
Venkatesh *et al*. (2011) 53	Y	Y	N	NR	N	NR	NA	Y	Moderate (42.9%)

Yes (Y). No (N). Not reported (NR). Not applicable (NA). ^a^The research question or objective was clearly described. ^b^The study population was explicitly specified. The article described the group of people from which the study participants were selected/recruited, using demographics, location and time period (i.e. who, where, when). Inclusion and/or exclusion criteria were clearly prespecified and applied uniformly to all participants. ^c^Participants (or clusters of participants) were selected as random cases. ^d^Participation rate was considered the percentage of eligible participants completing the study, and so analysed. If fewer than 50% of eligible individuals participated in the study, then there is concern that the study population does not adequately represent the target population. ^e^In order to determine whether an exposure causes an outcome, the exposure must come before the outcome. If a cohort study was conducted properly, the answer to this criterion should be “Yes.” In cross‐sectional studies (or cross‐sectional analyses of cohort studies), the exposures and outcomes were assessed during the same time frame. For cross‐sectional analyses, the answer should be “No.” ^f^The article clearly detailed how fertility desires/intentions (the outcome of interest) were measured (e.g. the specific question). The tools or methods to assess this outcome were objective or have been validated. The tools or methods reflected what they are supposed to measure. ^g^Usually, an acceptable overall follow‐up rate is considered 80% or more of participants whose exposures were measured at baseline. This criterion was only applicable for cohort studies. ^h^The potential confounding variables were measured and adjusted for. Logistic regression or other regression methods are often used to account for the influence of variables not of interest. Key factors that may be associated with both the exposure and the outcome should be controlled for in the analyses. ^i^The sampling procedure was described with detail in the article of Kidder *et al*. 83. ^j^Inclusion and/or exclusion criteria were not clearly prespecified. ^k^These data were reported in the article of Kuete *et al*. (2016) 48. ^l^The article of Mujugira *et al*. 84 described the design of the trial and the baseline characteristics of the Partners PrEP Study cohort. ^m^The article of Birungi *et al*. 85 described with more detail the inclusion and/or exclusion criteria and the participant source/selection.

**Table 4:** Risk of bias assessment of included mixed methods studies

Study (year) [Reference]	Qualitative (QL) component			Quantitative (QT) component			Mixed methods (MX) component		Rating
	Clear selection of participants^a^	Clear data collection^b^	Reported qualitative data analysis^c^	Sampling strategy^d^	Inclusion and/or exclusion criteria described^e^	Appropriate measurement of the outcome of interest^f^	Reported rational for integrating QL and QT methods^g^	Integration of QT and QL results relevant to address research question^h^	
Demissie *et al*. (2014) 56	Y	Y	Y	Y	Y	Y	U	Y	Strong (87.5%)
Klein *et al*. (2003) 65	Y	U	N	Y	Y	NA^i^	U	N	Moderate (42.9%)
Mindry *et al*. (2013) 66	U	Y^j^	Y	U	Y	Y	Y	Y	Strong (75%)
Myer *et al*. (2007) 57	Y	Y^j^	Y	Y	Y	Y	U	N	Strong (75%)
Rispel *et al*. (2011) 51	Y	Y	Y	Y	Y	NA^i^	Y	Y	Strong (100%)

Yes (Y). No (N). Unclear (U). Not applicable (NA). ^a^The selection/recruitment of participants was clear. ^b^The method of data collection (e.g. in‐depth interview, focus group) was clear and explicit (e.g. indication of how interviews were conducted). The form of the data (e.g. tape recordings, notes) was reported. ^c^Data analysis was stated and addressed the objective. ^d^A procedure for sampling was reported. ^e^Inclusion and/or exclusion criteria were explained. ^f^The outcome was clearly defined, and the measurement was appropriate for answering the research question. ^g^The rationale for integrating qualitative and quantitative methods to answer the research question (or objective) was explained. ^h^There was evidence that data gathered by both research methods were brought together to answer the research question. It was clear how and when integration occurred (during the data collection‐analysis and/or during the interpretation of qualitative and quantitative results). ^i^A qualitative method assessed the outcome of interest. ^j^However, the form of the data (e.g. tape recordings, notes) was not stated.

**Table 5:** Risk of bias assessment of included qualitative studies

Study (year) [Reference]	Clear statement of aim^a^	Qualitative methodology appropriate^b^	Reported recruitment strategy^c^	Data collection^d^	Ethical considerations^e^	Reported data analysis^f^	Clear statement of findings^g^	Research value^h^	Rating
Matthews *et al*. (2013) 58	Y	Y	Y	Y	Y	U	Y	Y	Strong (87.5%)
Ndlovu (2009) 59	Y	Y	Y	U	Y	Y	Y	Y	Strong (87.5%)

Yes (Y). Unclear (U). No (N). ^a^There was a clear report of the objectives of the research. ^b^The study sought to interpret or illuminate the actions and/or subjective experiences of participants. ^c^The article described how the participants were selected/recruited. ^d^Data were collected in a way that addressed the research question. It was clear how data were collected (e.g. in‐depth interview, focus group) and the researcher has made the methods explicit (e.g. indication of how interviews were conducted). The form of data (e.g. tape recordings, notes) was clear. ^e^The researcher has discussed issues around informed consent or confidentiality. Approval has been sought from the ethics committee. ^f^The analysis process was described, and it was reported how the categories/themes were derived from the data. ^g^The findings were explicitly reported and discussed in relation to the research question. ^h^The researcher discussed the contribution the study makes to existing knowledge or understanding (e.g. the study considered the findings in relation to current practice or policy, or relevant research‐based literature).

### 2.5 Analyses

We reported study findings and conducted a qualitative and descriptive analysis based on the reported outcomes. Each included study was synthesized according to the structured data extraction form previously described. Given the considerable heterogeneity across studies (e.g. study types/design; relevant findings), a meta‐analysis was not considered suitable.

## 3 Results and discussion

### 3.1 Study selection

**Figure 1:**
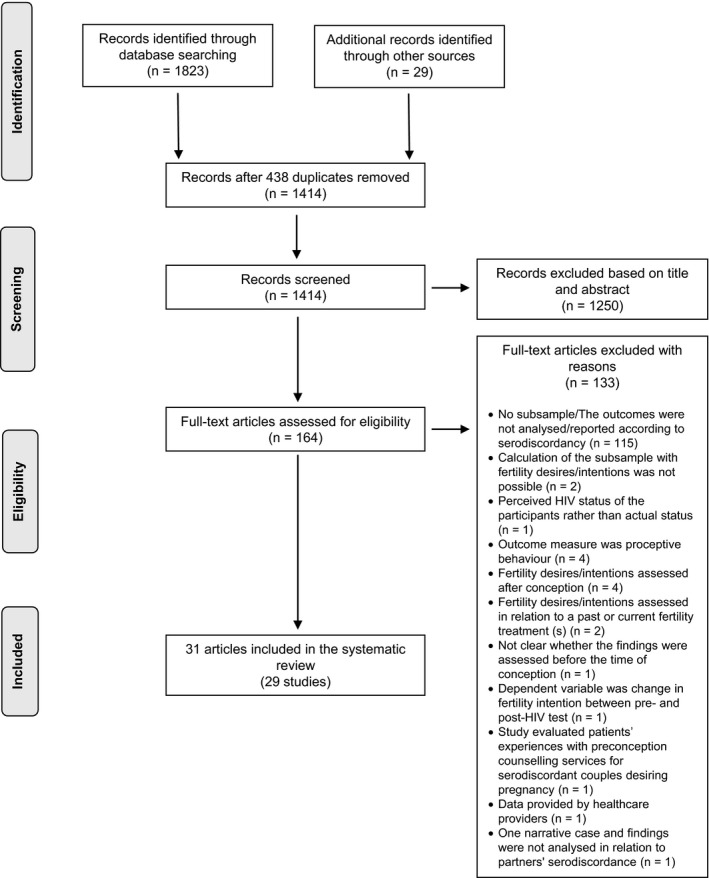
Flow chart of the article selection.

The search strategy identified 1852 records, from which we selected 164 eligible studies with available full‐texts (Figure 1). According to the review eligibility criteria, 133 papers were further excluded (see Figure 1 for detailed reasons). We contacted seven authors for clarification/further information. Five of these were excluded because they remained unclarified, and two were excluded after the authors’ clarification because they did not meet the eligibility criteria 44, 45. Because of overlapping samples, four articles (46 and 47; 48 and 49) were considered as two studies. Priority was given to the articles of Beyeza‐Kashesya *et al*. 47 and Kuete *et al*. 49. These studies were prioritized because they included both findings about the prevalence of fertility desires/intentions and the associated factors. Therefore, 29 different studies reported in 31 journal articles met all of the inclusion criteria and were included in the systematic review.

### 3.2 Study characteristics

Of the twenty‐nine studies, twenty‐one were conducted in LMIC (Table 1) and eight studies were conducted in HIC (Table 2). Regarding the studies conducted in LMIC, three were multi‐country studies (14.3%), all of which were sub‐Saharan African countries 24, 50, 51. Regarding the eighteen studies conducted in one country (85.7%), most were conducted in sub‐Saharan African countries (14/18; 77.8%), two studies in India 52, 53 and two in Brazil 54, 55. Most studies were quantitative (16/21; 76.2%), three used mixed methods 51, 56, 57 and two were qualitative 58, 59. Twenty studies had a cross‐sectional design (95.2%); one study reported a cohort design 1. The number of participants in serodiscordant relationships ranged from 4 to 1682 (M =* *271.05; SD = 430.05). Most studies included only one member of the dyad (n = 16; 76.2%). Five studies included both members of the dyad 47, 50, 51, 59, 60, and the number of serodiscordant couples ranged from 2 to 571 (M =* *226.40; SD = 250.67). Regarding the sex of the index partner, women were the most frequent partner living with HIV (14/17; 82.4%). In the five studies that included both members of the couple, women were the partner living with HIV in a higher percentage of couples 50, 51 or in all participating couples 59. In two studies, men were the most frequent partner living with HIV 47, 60. In four studies, the sex of the index partner was not reported 52, 56, 61, 62.

Ten studies (10/21; 47.6%) did not report information about the research question that specifically assessed fertility desires/intentions. Eight studies 22, 24, 47, 52, 56, 57, 60, 63 reported how they asked the question to participants, of which three clearly mentioned a binary response choice 52, 56, 63 and two a question with four response categories 47, 60. Two studies 49, 53 did not report the question(s) addressing fertility desires/intentions; however, they provided the operational definition. One study 50 did not clearly report whether the items enumerated were used to assess fertility desires/intentions.

In HIC, seven studies were conducted in the US (87.5%) and one in Switzerland 64. Six studies were quantitative (75%), and two were mixed methods studies 65, 66. Seven studies (87.5%) had a cross‐sectional design, one of which was a retrospective chart review 6. One study had a cohort design 67. The number of participants in serodiscordant relationships ranged from 22 to 286 (M* *=* *100.63; SD = 86.63). Most studies included only one member of the couple (5/8; 62.5%). Three studies included both members of the dyad 6, 65, 67, and the number of serodiscordant couples ranged from 11 to 143 (M =* *68; SD = 67.82). In the three studies with WLWH in a serodiscordant relationship, they were the index partner 25, 68, 69. In the three studies that included both members of the dyad, the man was the index partner 6, 65, 67. In two studies, this information was not reported 64, 66.

Almost all studies (7/8; 87.5%) reported how the fertility desires/intentions were assessed, of which four clearly reported a dichotomous response choice 6, 66, 68, 69 and one mentioned that responses greater than zero represented fertility desires/intentions 25. One study did not report any information about the question specifically assessing the outcome of interest 64.

### 3.3 Risk of bias within studies

Regarding the twenty‐two quantitative studies, twelve were rated as moderate quality (54.5%), seven as strong quality (31.8%) and three as poor quality (13.6%; Table 3). For all studies, the objective was clearly stated, and for most of them, the study population was clearly defined and eligibility criteria were described (81.8%), the participation rate was 50% or more (59.1%) and methods to control for confounding were used (68.2%). Eleven studies used appropriate measures for assessing the outcome of interest. The sample was representative in five studies (22.7%), and for two (the cohort studies), the exposure was assessed prior to outcome measurement (9.1%). For these cohort studies, the loss to follow‐up after baseline assessment was not reported. The percentage of agreement between the first and second authors was high (93.8%). The inter‐rater agreement was almost perfect (*k *=* *0.91, *p *< 0.001).

Of the five mixed methods studies, four were rated as having strong quality and one as moderate (Table 4). However, the mixed methods component was the weakest one. Only two studies clearly reported the rationale for integrating qualitative and quantitative methods. Inter‐rater agreement for the assessment of mixed methods studies was substantial (*k *=* *0.79, *p *< 0.001). The authors agreed on 90% of the criteria. The two qualitative studies were rated as strong quality (Table 5). Inter‐rater agreement for the assessment of qualitative studies was moderate (*k *=* *0.43, *p *=* *0.086), despite the high percentage of agreement (87.5%). Consensus was reached for all studies.

### 3.4 Low‐ and middle‐income countries

#### 3.4.1 Prevalence of fertility desires/intentions

Concerning PLWH with a partner living without HIV, in three studies, most participants in serodiscordant relationships reported high fertility desires/intentions (62% to 81%) 53, 61, 62. Also, among PLWH with a partner living without HIV, five studies presented percentages between 19% and 39% 24, 52, 55, 56, 57. For PLWH with a partner living without HIV or HIV unknown status partner, one study 58 revealed that 44% of the participants desired for child in future. Lastly, among PLWH with a partner with unknown HIV status, a wider range of percentages was observed in the four studies, varying from 8% to 63% 24, 52, 55, 61.

Regarding WLWH with a partner living without HIV, eight studies found percentages between 43% and 84% 1, 22, 49, 54, 57, 63, 70, 71. In one study, a similar proportion of HIV‐infected women (48%) and HIV‐uninfected women (42%) in serodiscordant couples reported desiring additional children 1. A lower prevalence of 27% was found among WLWH with a recent pregnancy with a partner living without HIV/partner with HIV unknown status 58. In this study, HIV‐infected men with an HIV‐uninfected/HIV unknown status partner presented a higher prevalence than HIV‐infected women (70%). In the other two studies 50, 57, the prevalence was higher among women (even if by a small difference).

Three studies that included both members of the couple found that most participants/individuals (49% to 59%) in these serodiscordant relationships reported fertility desires/intentions 47, 51, 59. The only study that considered the sex of the index partner (positive‐woman couples vs. positive‐man couples) 47 showed that more than half of the participants in both positive‐woman couples (64%) and positive‐man couples (55%) reported the desire to have children (the difference was not statistically significant). One study revealed a lower prevalence, with only 28% reporting wanting more children 60.

When the couple was the unit of analysis, two studies found a lower prevalence: for 16% 60 and 21% 50 of the couples, both members reported to desire/intend to have children. These two studies also assessed the agreement between partners of the dyad (i.e. if they both agreed in considering children or if they both agreed in not considering children) and found that most couples (64% in Muldoon *et al*. 60 and 76% in Mujugira *et al*. 50) agreed in relation to fertility desires/intentions. Ndlovu 59 found that for half of the couples, both members reported the desire to have children.

#### 3.4.2 Factors associated with fertility desires/intentions

Five studies assessed factors associated with fertility desires/intentions 47, 49, 50, 51, 58, but only one 47 considered the sex of the index partner. In one study 11, despite the inclusion of both members of the dyad, the analysis was only performed for the partners living with HIV.

Regarding individual factors, our findings indicated that a fewer number of living children 47, 49 or having no children 51 were associated with increased fertility desires/intentions. Matthews *et al*. 58 found that a higher number of living children was related to decreased desire for children in the future. In two studies 47, 50, younger age was associated with increased fertility desires/intentions. In two studies, factors related to ART were also recognized: expressing interest in early initiation of ART 50 and, among positive‐man couples, possessing the knowledge that ART is more than 70% effective in preventing vertical transmission of HIV 47. However, in two studies, being on ART was not associated with fertility desires/intentions 47, 51.

Regarding couple‐level factors, in three studies, factors within the couple's relationship were also associated with fertility desires/intentions: the belief that the partner wanted to have a child, irrespective of the sex of the index partner 47; having had discussions with the partner about when to get pregnant (among positive‐woman couples) 47; having no children with the current partner 50; having unprotected sex in the prior month 50; the fear of infecting the partner living without HIV and the partners’ conflicting desires 51.

In the structural domain, and concerning health systems, the lack of availability and affordability of alternatives to condomless heterosexual vaginal intercourse was recognized in one study as influencing the intentions in these resource‐limited settings 51. In one study 47, not having had discussions with health workers about contraception and HIV, among positive‐man couples, was associated with increased desire to have children; conversely, discussing with health workers about pregnancy and HIV was not associated with fertility desires. Yet, seeking medical professional advice was also mentioned as playing an important role in childbearing decisions 51. Factors related to the cultural context/norms and to perceived/experienced stigma were described in one study 47: pressure from relatives for the couple to have children; and, among positive‐woman couples, not having disclosed the serostatus to family and wanting HIV status to remain a secret.

### 3.5 High‐income countries

#### 3.5.1 Prevalence of fertility desires/intentions

One study found that 42% of PLWH with a partner living without HIV reported a current strong desire for children 64. Another study of PLWH with a partner living without HIV/HIV unknown status partner reported a prevalence of 58% 66. Regarding WLWH, the three studies found percentages varying from 32% to 55% 25, 68, 69.

All the studies with HIV‐serodiscordant couples in which the two members were included were positive‐man couples in the context of assisted reproduction. These studies revealed percentages between 64% and 73% 6, 65, 67. All studies presented results for both sexes and found high and very similar percentages between women (64% to 73%) and men (66% to 73%). Gosselin and Sauer's 6 study reported the intercouple agreement and found high agreement between female and male partners regarding the desire to have children (*k *=* *0.85, *p *<* *0.001).

#### 3.5.2 Factors associated with fertility desires/intentions

Factors were only reported in one study 6. This study revealed that couples who desired additional children in the future were more likely to be younger (individual factor), to not have children together, to have shorter relationship length and to have begun their relationship after the male partner's HIV diagnosis (couple‐level factors).

## 4 Discussion

This is the first systematic review synthesizing the literature on the prevalence of fertility desires/intentions and its associated factors among individuals in serodiscordant relationships, specifically distinguishing low‐ and middle from HIC. Most studies were classified with moderate/strong methodological quality, and a broad range of study types was considered, providing a comprehensive review of the literature in this area. The prevalence was especially heterogeneous in LMIC in comparison to HIC, as well as within LMIC. However, many individuals in HIV‐serodiscordant relationships reported desire/intention to have children. Few studies analysed the factors associated with fertility desires/intentions: younger age, a fewer number of living children or the absence of children with the partner were factors consistently associated with increased fertility desires/intentions.

Regardless of the country income level, most studies were conducted with PLWH or WLWH in a serodiscordant partnership rather than with serodiscordant couples. The results of 17 out of 29 studies showed that at least half of the participants had fertility desires/intentions. Comparing the fertility desires/intentions between individuals in serodiscordant relationships and PLWH in general, the prevalence was higher among those in serodiscordant relationships [e.g. 71]. Studies that compared individuals in serodiscordant relationships with those in seroconcordant partnerships have also found that participants with a partner living without HIV were more likely to report fertility desires/intentions in comparison to those with a partner living with HIV [e.g. 56, 68, 71]. These findings support the relevance of promoting among healthcare providers the assessment of fertility desires/intentions of serodiscordant couples and informing these couples about how to conceive safely 72.

In HIC, the results showed lower variability in the prevalence of fertility desires/intentions (32% to 73%). However, in this setting, a higher prevalence (64% to 73%) was observed in studies that were all conducted in the same country (US), with couples (both members included), in which the man was the index partner, and in the context of assisted reproduction 6, 65, 67. The fact that these couples were seeking fertility treatment, and thus, all had an interest in conceiving a child may explain these high percentages. In LMIC, a greater heterogeneity of results was observed (8% to 84%), even between sub‐Saharan African countries. The prevalence of fertility desires/intentions seems to be distinct as the regions themselves; even in the same country, the prevalence was found to vary. In two studies conducted in Uganda 47, 60, with both members of the couple included (mutually disclosed) and in which the man was the most frequent index partner, revealed a prevalence as different as 59% 47 and 28% 60. The rationale for these differences was not clear, although, as it was suggested by Demissie 56 and Melaku *et al*. 63, they are probably related to specific sociodemographic/economic/cultural characteristics in each country or region of the country. For example, in Nigeria, according to Iliyasu *et al*. 61, despite the elimination of cost of HIV medications in government hospitals, differences in the use of health services still exist between the poor and the wealth, as well as between urban and rural areas. Furthermore, the fear of stigma and discrimination by communities and healthcare providers can prevent individuals from accessing health services in their community, and, consequently, choosing more distant centres 61. These differences may also be explained by specificities of the study samples (e.g. age of participants; if they had other children) and/or data collection as well as different operationalization of fertility desires/intentions or their method of assessment. However, in LMIC, because almost half of the studies did not report information about the question that specifically assessed fertility desires/intentions, it was not possible to draw definite conclusions.

In serodiscordant relationships, only few studies analysed the factors associated with fertility desires/intentions and only one was conducted in HIC 6. At an individual level, our findings showed that individuals in serodiscordant relationships with a younger age 6, 47, 50 and a fewer number of living children/having no children 47, 49, 51, 58 may be more likely to desire/intend to have children. Couples (particularly, positive‐woman couples) in these circumstances may be those who are most pressured by relatives to have children, particularly in LMIC, where the family is often part of the decision‐making process and may not know about the infection 47. Indeed, as suggested by the social ecological framework 33, factors from the structural domain (e.g. cultural context/norms) may interact with individual factors. Other important factors at the structural level should be noted, such as disclosure to family 47 and discussion/counselling with healthcare providers 47, 51. Among positive‐woman couples, those who did not disclose their HIV status to relatives and that wanted to remain it a secret reported an increased desire/intention to have children. Particularly, women may consider childbearing in order to conceal their HIV‐positive status and to introduce a sense of “normality” to their lives, avoiding HIV‐related stigma and discrimination from the family and the community 73, 74. Discussions with health workers showed mixed results; discussions about childbearing was not associated with fertility desires/intentions in one study 47, but in another, the information provided by medical personnel was considered important 51. It is crucial to understand the perceptions that couples have regarding healthcare providers attitudes (e.g. if they perceive that they will be stigmatized), once they can have a unique role supporting individuals/couples in the decision‐making process, while reducing the likelihood of HIV transmission 46.

Our review indicates that being on ART was not associated with fertility desires/intentions 47, 51, which was also demonstrated in a previous meta‐analysis 28. However, expressing interest in early initiation of ART 50 and, specifically among positive‐man couples, possessing the knowledge that ART is highly effective in reducing mother‐to‐child transmission 47 were factors associated with increased fertility desires/intentions. ART has been consistently associated with improvements in physical wellbeing and perceived quality of life 75, 76, and therefore may impact the desire/intention to have children; however, these studies 47, 51 were conducted before the publication (in 2011) of the landmark finding that early initiation of ART (the most recent guidelines recommend immediate initiation 77) was associated with a 96% lower risk of HIV seroconversion within serodiscordant couples 78. Therefore, participants of those studies could not have been expected to know the importance or rely on treatment as prevention. Nevertheless, these findings warrant further in‐depth investigation, especially in countries where access to ART is especially unevenly distributed 79. Additionally, it would be important to understand if nowadays the association between being on ART and fertility desires/intentions would be different, considering that empirical research has strongly supported that PLWH who are on ART and whose level of HIV is suppressed to undetectable levels will not transmit HIV sexually [e.g. 15].

Despite scarcely examined, some couple‐level factors also emerged. In line with the individual factor concerning the number of children/having children, those who did not have children with their partner showed increased fertility desires/intentions 6, 50. The belief that the partner wanted to have a child was considered the major determining factor 47. This finding is congruent with other findings that have shown the influence of the partner on fertility decision‐making 72, 80, 81. It may be important to note that in opposite‐sex couples man has often a greater decision‐making power within the couple 33, and therefore, when assessing only the couple, the results may only reveal his preferences/choices. For instance, one study concluded that male preferences were more influential when the individual desires differed 20. The fear of infecting the partner living without HIV 51, having begun relationship after the male partner had already been diagnosed, and a shorter relationship length 6, 24, 56 were also factors identified in different studies.

Some limitations at the studies and review levels should be noted. The studies included in this review were conducted mainly in sub‐Saharan African countries, where most of serodiscordant couples are thought to be concentrated 3. The studies from HIC were all (except one) conducted in the US. Therefore, studies in more diversified HIC are necessary to better compare the challenges faced by serodiscordant couples in different economies and to examine whether cultural differences/economic background influence fertility desires/intentions. Additionally, most of the associated factors were identified in a minority of studies, mainly in LMIC, which difficult to generalize these findings. Regardless of the country income level, the number of studies involving both members of the serodiscordant dyad was very low (8/29) and most studies relied on the responses of a single partner. Given the centrality of interpersonal dynamics within a relationship, without partner's data, it is not possible to determine the extent to which one partner may inflate the other partner's desire/intention based on their own desire/intention. If couples‐based approaches are to be employed within HIV prevention, more studies focused on the couple as the unit of analysis are needed 2. In this review, studies with partners of any sexual orientation were included; however, the comparison of opposite‐sex versus same‐sex relationships was not possible. Two reasons may account for this: studies included both participants in opposite‐sex and same‐sex couples, although the results were analysed in general [e.g. 51]; or studies did not consider this as an inclusion/exclusion criterion and did not clearly specify whether the individuals were in opposite‐sex or same‐sex relationships. Despite the increasing visibility of non‐heterosexual parenting 2, 82, our findings showed that discussions about fertility in the context of HIV happened almost exclusively in relation to opposite‐sex relationships.

The terms desires and intentions were used interchangeably throughout articles [e.g. 22, 50, 53, 60, 71] or simultaneously in the same question 68. This lack of uniformity within and between studies may represent a lack of clarity and hinder the interpretation of the findings. For only five studies, the sample was considered representative, and in some studies [e.g. 59, 66], the number of participants in serodiscordant relationships was low. Thus, the results should be interpreted with caution. Most studies were cross‐sectional, which precludes causal and temporal relationships. Because decision‐making is a process and decisions about fertility may change over time 47, longitudinal studies would be valuable. In most studies, despite the method of assessment of the research question was fairly adequate, many studies from LMIC did not clearly state the question specifically addressing fertility desires/intentions. This could be important to explain (at least partially) the variability in results found for the prevalence in LMIC.

At the review level, first, only one researcher screened the titles and abstracts of the electronic and reference list searches, which may result in potentially missed studies or biased exclusion of articles. Second, our definition of serodiscordant couples/partners included partners with unknown HIV status, which may be infected. However, counselling to both individuals in the context of a relationship with a partner living without HIV or a partner with unknown HIV status may be important in terms of prevention to reinforce the importance of routinely being tested for HIV. Third, we considered studies conducted in the context of assisted reproduction. Despite we only included studies in which fertility desires/intentions were assessed in relation to future/additional children after the initial assisted reproduction treatment, the prevalence in HIC should be interpreted considering this specific context. Fourth, not including grey literature as well articles published in languages other than English may have introduced publication bias. Fifth, we were unable to pool the data for meta‐analysis because of the significant heterogeneity across studies.

## 5 Conclusions

Based on this review, it is reasonable to conclude that being in an HIV‐serodiscordant relationship does not stop individuals from desiring or intending to have children. Policy makers, programme implementers and clinicians working with PLWH should pay particular attention to individuals in serodiscordant relationships who are younger and those who have yet to have children or who have few children. Furthermore, despite sparse, different couple‐level factors were found to be associated with fertility desires/intentions, suggesting the importance of analysing this topic also in the context of an intimate relationship.

Potential interventions that can be implemented in this area should also consider the multiple‐levels highlighted by the social ecological framework and how they are interlinked 34, as well as the economic context of individuals/couples. Indeed, the economic context may shape access to ART, PrEP and medically assisted reproduction, and consequently, influence individual‐level resources that can facilitate access/adherence to these interventions. Social norms around gender (structural domain) may also shape interactions between individuals in serodiscordant couples (couple‐level) and individual self‐efficacy to engage in discussions about this topic and make informed decisions. Accordingly, including men in discussions with their partners on issues related to safer conception strategies may help change these dynamics 33, 34. This reinforces the importance of considering both the individual and the dyad. Given the mutual impact that members of a dyad have on each other's lives, the inclusion of both partners in the discussions about fertility and safer conception practices may be a more effective strategy to respond to their reproductive needs 72. However, it may be important to not forget some challenges when including both members of the couple in these interventions. For example, it may be difficult for the partner living without HIV to attend clinical visits at HIV clinics or to implement some safer conception strategies when partners are not mutually disclosed.

## Competing interests

We declare that there are no conflicting interests.

## Authors’ contributions

AM defined and conducted the search strategy, reviewed the titles and abstracts of the electronic and reference list searches, and assessed the studies for eligibility. AM analysed each article that met the inclusion criteria and extracted the required data, and SA checked these data. AM and SA independently assessed the risk of bias of the included studies. Any disagreement was discussed and resolved by consensus or, if necessary, by discussion with referral with MP, who supervised this process. AM wrote the first draft of the manuscript. CC and MCC assisted with all other authors mentioned and reviewed, edited and commented on all subsequent drafts of the manuscript, including the final draft. All authors have read and approved the final manuscript.

## Additional files 1

### Additional file 1.

#### Search strategy used in PsycINFO

**Table Additional file 1:** Example of search strategy used in PsycINFO via OvidSP (modified as needed for use in the other databases)

No.	Search term	Results
1	Human immunodeficiency virus.tw.	5652
2	Human immune deficiency virus.tw.	27
3	HIV.tw.	44899
4	exp*HIV/	32932
5	HIV‐1.tw.	2014
6	HIV‐2.tw.	77
7	Acquired Immunodeficiency Syndrome.tw.	724
8	Acquired Immune deficiency Syndrome.tw.	3048
9	AIDS.tw.	35002
10	exp*AIDS/	11531
11	1 or 2 or 3 or 4 or 5 or 6 or 7 or 8 or 9 or 10	60993
12	Fertility desire*.af.	313
13	Fertility intention*.af.	473
14	Reproductive intention*.af.	238
15	Reproductive decision making.af.	527
16	Desire to have children.af.	225
17	Desire for child.af.	25
18	Childbearing desire*.af.	97
19	Childbearing intention*.af.	134
20	Parenthood.af.	18910
21	exp PARENTHOOD STATUS/	2259
22	Fatherhood.af.	7464
23	Motherhood.af.	19445
24	Paternity.af.	5757
25	Maternity.af.	8807
26	12 or 13 or 14 or 15 or 16 or 17 or 18 or 19 or 20 or 21 or 22 or 23 or 24 or 25	50664
27	Relationship.af.	898861
28	Couple.af.	35337
29	exp COUPLES/	12230
30	Married.af.	40493
31	Marriage.af.	114338
32	exp MARRIAGE/	10442
33	Partner.af.	83546
34	Partners.af.	93042
35	Spouse.af.	26607
36	exp SPOUSES/	14925
37	Dyad.af.	12928
38	exp DYADS/	5178
39	Husband.af.	11137
40	Wife.af.	17518
41	Discordant.af.	11691
42	Discordants.af.	5
43	Discordancy.af.	101
44	Discordance.af.	4100
45	Serodiscordance.af.	110
46	Serodiscordancy.af.	3
47	Serodiscordant.af.	1170
48	Serodiscordants.af.	4
49	Sero‐discordant.af.	125
50	Sero‐discordants.af.	0
51	Sero‐discordance.af.	7
52	Sero‐discordancy.af.	0
53	27 or 28 or 29 or 30 or 31 or 32 or 33 or 34 or 35 or 36 or 37 or 38 or 39 or 40 or 41 or 42 or 43 or 44 or 45 or 46 or 47 or 48 or 49 or 50 or 51 or 52	1063935
54	11 and 26 and 53	900

Searches were performed using OvidSP, in which “.af.” represents all fields, that simultaneously search in all searchable fields in the database, “.tw.” represents text word field, that is an alias for all of the fields in the database that contain text (in PsycINFO include table of contents, title, abstract and key concepts), “exp” represents explode, that expands the search results of terms entered and include more specific related terms; “*” preceding the indicated word (i.e. *<term>) represents a main topic; and “*” after the indicated word (i.e. <term>*) represents truncation, retrieving all possible suffix variations of the root word mentioned.

### Additional file 2.

#### Eligibility criteria: Exclusion criteria

Studies were not eligible for inclusion if: (1) they reported non‐original research (e.g. article reviews, meta‐analyses, discussion articles); (2) they were book chapters, unpublished studies, unpublished university dissertations, abstracts, communications, case studies, case reports or ongoing studies; (3) no outcomes of interest were reported; (4) it was impossible to compute or extrapolate the necessary data from the published results (e.g. the size of the sample/subsample to calculate the fertility desires/intentions prevalence); (5) the outcome measure was proceptive behaviour (i.e. behaviour that is designed to achieve conception, often measured by asking participants if they were trying to have children/get pregnant); (6) the outcome measure was exclusively pregnancy or pregnancy‐related decisions (to maintain a pregnancy vs. to terminate a pregnancy or to get sterilized); (7) fertility desires/intentions were assessed in relation to a past or current fertility treatment(s) in the context of assisted reproduction services when the persons who resorted to these treatments were already trying to achieve pregnancy (if fertility desires/intentions were assessed in relation to future/additional children after the treatment(s), the studies were considered). We opted to focus the review by excluding citations that were not written in English and that were conducted in the precombination ART era (i.e. before 1996).
